# Optimizing Clinical Temporal Relation Extraction With Large Language Models: Comparative Analysis

**DOI:** 10.2196/89957

**Published:** 2026-10-15

**Authors:** Jianping He, Zenan Sun, Laila Rasmy, Haifang Li, Jianfu Li, Evan Yu, Degui Zhi, Cui Tao

**Affiliations:** 1 McWilliams School of Biomedical Informatics, The University of Texas Health Science Center at Houston, Houston, TX, United States; 2 Department of Artificial Intelligence and Informatics, Mayo Clinic in Florida, 4500 San Pablo Rd S, Jacksonville, FL, 32224, United States, 1 904 956 3256

**Keywords:** clinical temporal relation extraction, large language model, fine-tuning, prompt tuning, low-rank adaptation

## Abstract

**Background:**

Clinical temporal relation extraction (CTRE) is essential for reconstructing patient timelines from unstructured electronic health records (EHRs). However, the linguistic complexity of clinical notes and the high cost of expert annotation impede the development of large-scale training corpora. While large language models (LLMs) have transformed general natural language processing, their application to CTRE remains underexplored.

**Objective:**

This study aimed to determine the optimal adaptation strategy for CTRE by conducting a controlled comparative benchmarking of LLM architectures and fine-tuning methodologies in both data-rich and limited-data regimes.

**Methods:**

We evaluated 4 LLMs representing 2 distinct architectures—transformer encoders (GatorTron [University of Florida and NVIDIA]) and transformer decoders (LLaMA 3.1-8B [Meta AI] and MeLLaMA-13B [Yale University and University of Florida]). We compared four adaptation strategies: (1) standard fine-tuning, (2) hard-prompting, (3) soft-prompting, and (4) low-rank adaptation (LoRA). Experiments were conducted on the 2012 i2b2 CTRE benchmark in both full-supervision and 1-shot scenarios.

**Results:**

Recent LLMs combined with appropriate adaptation strategies achieved strong performance for CTRE on the 2012 i2b2 benchmark. In the data-rich setting, the best-performing configuration was LLaMA3.1-8B with quantized low-rank adaptation (QLoRA) applied to the query and value projection layers, achieving a micro-*F*_1_-score of 89.15% (0.30). Comparative analyses showed that QLoRA applied only to the query and value projection layers achieved performance comparable to QLoRA applied to all linear layers, while requiring fewer trainable parameters. Hard-prompting showed particular effectiveness in the 1-shot scenario compared with standard fine-tuning for GatorTron-Base. Overall, Decoder-based models generally outperformed encoder-based models under QLoRA-based adaptation. However, under the most resource-constrained setting, where both training data and trainable parameters were highly limited, encoder-based models achieved better performance than decoder-based models.

**Conclusions:**

This study provides a controlled benchmark for adapting LLMs to CTRE under different data and parameter constraints. Our findings suggest that QLoRA targeting only the query and value projection layers is a computationally efficient and competitive adaptation strategy. Decoder-based LLMs generally achieved stronger performance under QLoRA-based adaptation, whereas domain-specific encoder-based models showed advantages under the most resource-constrained soft-prompting setting. These results highlight the importance of selecting adaptation strategies according to both model architecture and available computational resources.

## Introduction

### Background and Significance

In recent years, temporal relation extraction (TRE) has attracted growing interest in clinical research due to its crucial role in understanding disease progression, drug efficacy, and patient outcomes [1]. Clinical temporal relation extraction (CTRE) aims to extract the temporal information about the patient’s past, current, and future status or conditions that are unavailable in structured electronic health records (EHRs) data [2,3]. This process is essential for reconstructing the chronological understanding of the events within clinical documents [1,4]. Accurate recognition and extraction of temporal relations between clinical events are crucial in improving diagnostic accuracy and informing treatment strategies [4].

However, extracting this information is uniquely challenging. Clinical notes have flexible formatting [5,6]. Therefore, medical providers prefer to use atypical grammatical constructions, including a lot of abbreviations and acronyms in clinical notes [7,8], which makes the annotation very troublesome in CTRE [9]. The CTRE annotation has a lower interannotator agreement (IAA) than other clinical annotation tasks, including clinical events and temporal mentions annotation tasks [3]. Consequently, constructing high-quality, large-scale datasets for supervision is difficult, creating an urgent need for systems that can achieve robust performance even in few-shot learning environments.

### Evolution of Methods

Historically, the methodologies adopted in CTRE include rule-based methods, machine learning-based methods, deep learning-based methods, and hybrid methods [1]. The early rule-based systems for CTRE primarily relied on hand-crafted linguistic rules and domain-specific knowledge [10,11]. The machine learning methods used in CTRE consist of conditional random fields (CRF) [12,13], support vector machines (SVM) [14], random forests (RF) [15], and logistic regression (LR) [16]. Deep learning-based methods consist of recurrent neural networks (RNN) [17], convolutional neural networks (CNN) [18], long short-term memory (LSTM) [19], and BERT variants [20]. Deep learning systems or hybrid systems tend to outperform other methods [1]. Advances have been predominantly driven by Transformer-based architectures, particularly BERT variants, which excel at modeling long-range dependencies [20-24]. Despite these methodological improvements, the current state-of-the-art (SOTA) CTRE performance remains suboptimal and poses limitations for downstream clinical applications [1].

Furthermore, large language models (LLMs) have demonstrated exceptional efficacy across a diverse spectrum of natural language processing (NLP) tasks, driven by their advanced semantic understanding and generative capabilities [25-27]. In the biomedical domain, models such as GatorTron (University of Florida and NVIDIA), LLaMA 3.1 (Meta AI), and MeLLaMA (Yale University and University of Florida) have achieved notable advancements in information extraction, excelling at the identification, classification, and normalization of clinical entities within unstructured health care data [26,28,29]. However, despite their proven utility in general clinical NLP, to the best of our knowledge, the application of these advanced architectures to the specific complexities of the 2012 i2b2 CTRE task remains underexplored.

### The Research Gap

While recent studies have begun to apply LLMs to CTRE, significant gaps remain. For instance, Peng et al [28] explored prompt tuning for GatorTron but did not evaluate parameter efficient fine-tuning (PEFT) methods like low-rank adaptation (LoRA) or benchmark performance on the i2b2 2012 dataset. Similarly, while decoder-based models have been tested in recent shared tasks [30,31], these studies often rely on zero-shot evaluations or fail to investigate the newest SOTA models, such as LLaMA 3.1. To our knowledge, no study has yet comprehensively compared the performance of medical-specific encoders (eg, GatorTron) against general and medical decoders (eg, LLaMA 3.1 and MeLLaMA) using a unified set of fine-tuning strategies.

### The Proposed Study

To bridge these gaps, this study systematically benchmarks fine-tuning strategies for adapting LLMs to CTRE, evaluating performance across both data-rich (full supervision) and limited-data (few-shot) regimes on the 2012 i2b2 CTRE task [32]. Acknowledging the prohibitive memory costs associated with full-parameter optimization [33], we contrast standard fine-tuning and hard-prompting [34] with resource-efficient PEFT alternatives, specifically soft-prompting [35] and LoRA [36].

We benchmark following 4 distinct LLMs: GatorTron-Base (encoder-based, medical), GatorTron-Large [37] (encoder-based, medical), LLaMA 3.1 [38,39] (decoder-based, general), and MeLLaMA [26] (decoder-based, medical). By systematically applying standard fine-tuning, hard-prompting, soft-prompting, and LoRA, we aim to provide a roadmap for selecting the optimal architecture and tuning strategy for CTRE.

### Research Questions

Specifically, this study seeks to answer the following research questions:

1. Strategy efficiency: Does hard-prompting outperform standard fine-tuning in model performance?
2. Scaling laws: Does increasing the number of trainable parameters consistently lead to improved CTRE accuracy?
3. Architecture comparison: Given a comparable number of trainable parameters, does an encoder-based or decoder-based architecture demonstrate superior performance?
4. Resource constraints: Can soft-prompting achieve competitive performance with a highly constrained parameter budget?

## Methods

### Dataset

In this study, we used the 2012 i2b2 Challenge dataset on temporal relations in clinical narratives [32] to assess the performance of our proposed fine-tuning strategies. Table 1 provides a summary of the i2b2 temporal relation corpus. The dataset comprises 310 anonymized patient discharge summaries, containing over 178,000 tokens, annotated for clinically relevant events (EVENT), temporal expressions (TIMEX3), and their relationships. Each discharge summary, on average, contains 86.6 annotated events, 12.4 temporal expressions, and 176 temporal relations.

The corpus categorizes 8 types of temporal relationships among events and temporal markers: BEFORE, AFTER, SIMULTANEOUS, OVERLAP, BEGUN_BY, ENDED_BY, DURING, and BEFORE_OVERLAP. Due to low IAA on certain temporal relationships, the challenge organizers established a consolidated taxonomy for evaluation purposes. In this official schema, the 8 relations were merged into three broad categories: (1) BEFORE (encompassing BEFORE, ENDED_BY, and BEFORE_OVERLAP); (2) AFTER (encompassing AFTER and BEGUN_BY); and (3) OVERLAP (encompassing SIMULTANEOUS, OVERLAP, and DURING). To ensure comparability with previous benchmarks, our study evaluates model performance exclusively on these 3 consolidated classes.

CTRE can be classified into 2 types; one is DocTimeRel, which refers to the temporal relations between the document creation time (DCT) and the events; the other one is temporal link (TLINK), which refers to the relations between the mentions (clinical events or temporal expressions) over text [1]. Among all temporal relations in the 2012 i2b2 CTRE test set, 48.01% (13,317/27,736) are EVENT-section time temporal relationships. The remaining ones are nonsection time TLINKs. Among all temporal relationships, 42.37% (11,752/27,736) are EVENT-EVENT temporal relationships, 7.34% (2035/27,736) are EVENT-TIMEX3 temporal relationships, 0.77% (214/27,736) are TIMEX3-TIMEX3 temporal relationships, 1.51% (418/27,736) are TIMEX3-EVENT temporal relationships [40]. Within the category of nonsection time TLINKs, OVERLAP relations constitute 63.48% (9153/14,419), BEFORE relations make up 21.93% (3162/14,419), and AFTER relations represent 14.59% (2104/14,419) of the total nonsection time TLINKs [40].

**Table 1.** Summary of the 2012 i2b2 temporal relation corpus.

	Training set	Test set
Discharge summaries	190	120
Events	16,468	13,594
Temporal expressions	2366	1820
Temporal relations	33,543	27,736
BEFORE	17,513	15,113
AFTER	3207	2729
OVERLAP	12,823	9894

### Data Preprocessing

The original training set was further split into training and validation subsets at a ratio of 9:1, while the original test set was retained as the final evaluation set.

We adopted the same preprocessing methodology as outlined in the previous work by Chen et al [21]. The process begins by segmenting the discharge summaries into individual sentences. Then, for each temporal relation instance, we constructed a localized contextual input based on the positions of the 2 relation arguments. Specifically, we identified the sentence containing the subject argument and the sentence containing the object argument. If both arguments appeared in the same sentence, that sentence was used as the input context. If the 2 arguments appeared in different sentences, the complete sentence span between the 2 argument-containing sentences was retained as the input context. No fixed maximum number of sentences was imposed; instead, the context window was determined by the minimal contiguous sentence span containing both arguments. This strategy preserved the local discourse necessary for both intrasentence and intersentence temporal relation classification while reducing unrelated document-level information.

To explicitly indicate the target argument pair, the subject and object mentions were enclosed with special markers, [@Subject$]...[@Subject$] and [@Object$]...[@Object$], respectively. These markers were retained in all input formats used for each fine-tuning strategy, including standard fine-tuning, hard-prompting, soft-prompting, and quantized low-rank adaptation (QLoRA), so that the target argument pair was explicitly indicated to the model. For the hard-prompting strategy, the prompt template used in this study was “Subject [MASK] [MASK] Object.” The marked contextual input was combined with this prompt template, where the subject and object markers explicitly indicated which entity mentions corresponded to the subject and object positions in the template.

For encoder-based models, the maximum sequence length was set to 512 tokens, whereas for decoder-based models, it was set to 4096 tokens. The final model input was tokenized and truncated to the corresponding maximum length if it exceeded the model-specific limit.

### LLMs Used

This study uses 4 LLMs—GatorTron-Base (345M), GatorTron-Large (8.9B) [37], LLaMA3.1-8B [38,39], and MeLLaMA-13B [26].

#### GatorTron

GatorTron adopts a BERT-style architecture and was trained from scratch using 2 self-supervised tasks: masked language modeling (MLM) and sentence-order prediction (SOP). GatorTron was pretrained on over 90 billion words of text, including more than 82 billion words of deidentified clinical text. The GatorTron models were evaluated on 5 clinical NLP tasks spanning various linguistic levels—phrase, sentence, and document—using 6 publicly available benchmark datasets. Experimental results indicate that GatorTron outperformed existing biomedical and clinical transformers across all 5 tasks and 6 benchmark datasets. The model is available in three variants: GatorTron-Base (345M), GatorTron-Medium (3.9B), and GatorTron-Large (8.9B) [37].

#### LLaMA3.1

LLaMA3.1 was released by Meta AI in 2024. The pretraining process of LLaMA3.1 used more than 15 trillion tokens, all sourced from publicly available datasets [38,39]. One of the model’s most significant enhancements is the use of a tokenizer with a 128,256 token vocabulary, which enables more efficient language encoding. Additionally, LLaMA3.1 uses grouped query attention (GQA) to further optimize performance. LLaMA3.1 models, specifically the 8B, 70B, 405B, demonstrated superior performance across a wide range of tasks compared with other models of similar parameter scales. Notably, the LLaMA3.1-8B outperformed models of similar sizes, such as “Gemma2-9B” and “Mistral-7B.” The model was pretrained on sequences of 8192 tokens with a masking mechanism to ensure that self-attention does not cross document boundaries.

#### MeLLaMA

Xie et al [26] developed the MeLLaMA base models by continually pretraining LLaMA2 models using a mixed pretraining dataset comprising 129 billion tokens. The MeLLaMA-chat models were subsequently created by fine-tuning the MeLLaMA base models with an instruction tuning dataset containing 214,000 samples. Furthermore, leveraging a new medical evaluation benchmark (MIBE), which encompasses 6 critical medical tasks and 12 datasets, the authors fine-tuned the MeLLaMA base models with task-specific training sets from various evaluation datasets to assess their performance in a supervised learning setting. They also evaluated the Me-LLaMA-chat models in zero- and few-shot settings. MeLLaMA represents one of the most comprehensive open-source medical foundation LLMs, leveraging both biomedical and clinical data. Its evaluation demonstrates superior performance across a range of general and medical tasks when compared with other open-source medical LLMs, making it a highly promising option for applications in medical AI. MeLLaMA consists of 4 versions: MeLLaMA-13B, MeLLaMA-70B, MeLLaMA-13B-chat, and MeLLaMA-70B-chat.

To investigate the optimization of LLMs in resource-constrained scenarios—characterized by data scarcity and limited computational capacity—we focused on models with lower parameter counts, specifically, LLaMA3.1-8B and MeLLaMA-13B. To ensure a fair comparison across architectures of similar scale, GatorTron-Large (8.9B) was selected to represent the medical encoder-based architecture. Additionally, to thoroughly assess full-parameter-fine-tuning (FPFT) strategies, the smaller GatorTron-Base (345M) was included. Consequently, our comparative evaluation encompasses 4 models: GatorTron-Base (345M), GatorTron-Large (8.9B), LLaMA3.1-8B, and MeLLaMA-13B [26,37-39].

Table 2 illustrates the key architectural distinctions of various LLMs. GatorTron uses a transformer encoder-only design, processing raw text by tokenizing it into IDs using a 50,176-token vocabulary. These IDs pass through an embedding layer with a maximum sequence length of 512, producing embeddings with a 1024 feature dimension in the Base version and 3584 in the Large version. These embeddings then go through 24 (Base) or 56 (Large) transformer encoder layers, which generate contextualized representations by capturing intertoken relationships. Conversely, LLaMA3.1-8B and MeLLaMA-13B use a transformer decoder-only structure. Both models tokenize input text into IDs with vocabularies of 128,256 (LLaMA3.1-8B) and 32,000 (MeLLaMA-13B). These IDs are embedded into vectors with sequence lengths of 8192 and 4096, and feature dimensions of 4096 and 5120, respectively. The embeddings proceed through 32 (LLaMA3.1-8B) or 40 (MeLLaMA-13B) decoder layers, refining token contexts.

**Table 2.** The primary architectural differences among different LLMs^a^.

Models	Model architecture (layers)	Vocabulary size	Max sequence length	Feature dimension
GatorTron-Base	Encoder (24)	50,176	512	1024
GatorTron-Large	Encoder (56)	50,176	512	3584
LLaMA3.1-8B	Decoder (32)	128,256	8192	4096
MeLLaMA-13B	Decoder (40)	32,000	4096	5120

a LLM: large language model.

### Fine-Tuning Strategies for LLMs

#### Fine-Tuning Overview

Fine-tuning strategies include standard fine-tuning, hard-prompting, and PEFT. Standard fine-tuning is a supervised learning approach where the entire set of pretrained model parameters is refined. Hard-prompting appends fixed textual prompts to the input and reformulates the downstream task as a MLM problem, aligning it with the format of the pretraining task to improve the performance of language models [34]. Hard-prompting in transformer encoder-based models has demonstrated strong performance in few-shot scenarios across various NLP tasks [41-45]. Based on these findings, we hypothesize that applying this strategy to the CTRE task could significantly enhance performance, particularly in data-scarce settings. Both standard fine-tuning and hard-prompting necessitate full-parameter optimization, which results in prohibitive memory costs [33]. PEFT, also referred to as parameter efficient transfer learning or delta tuning, focuses on training only a small subset of parameters, either selected from the existing model or newly introduced [46-49]. This approach significantly reduces memory and storage demands since only the modified parameters are updated and saved. PEFT uses various techniques, including (1) adapter training integrating a bottleneck feed-forward network within each transformer block to enhance efficiency [50]; (2) LoRA identifying a low-rank delta within specific parameter matrices to optimize training [36]; and (3) soft-prompting appending a trainable matrix, known as a soft-prompt, to the input embeddings [35]. While soft-prompting may not be the most performant PEFT method, it is highly regarded for its exceptional parameter efficiency.

The fine-tuning strategies used in this study include (1) standard fine-tuning, (2) hard-prompting, (3) soft-prompting, and (4) LoRA. Additionally, quantized loading techniques are used to enhance computational efficiency. Standard fine-tuning and hard-prompting are full parameter fine-tuning strategies (Figure 1), whereas soft-prompting and LoRA fall under PEFT strategies (Figure 2).

**Figure 1.**
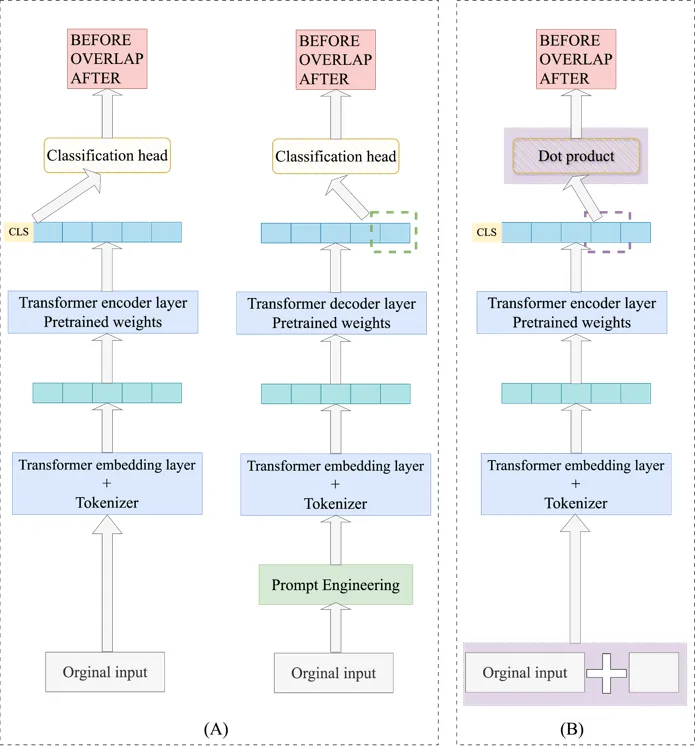
The principle of the implemented full parameter fine-tuning strategies. (A) Standard fine-tuning and (B) hard-prompting.

**Figure 2.**
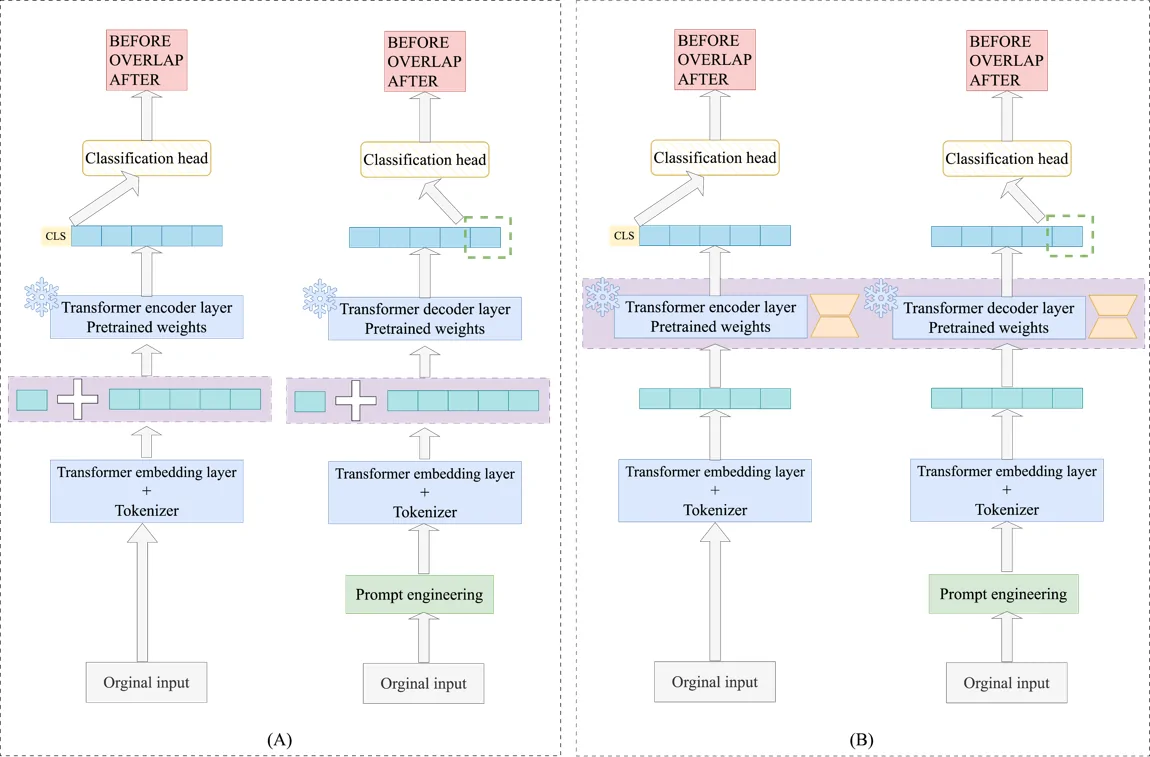
The principle of the implemented parameter-efficient fine-tuning strategies. (A) Soft-prompting and (B) low-rank adaptation (LoRA). The snowflake symbol indicates frozen model parameters. In soft-prompting, the backbone transformer layers remain frozen, whereas the continuous prompt embeddings concatenated with the static input embeddings are trainable. In LoRA-based adaptation, the backbone transformer layers remain frozen, and only the inserted LoRA modules are updated during training. The classification head is trainable in both settings.

#### Standard Fine-Tuning

Standard fine-tuning is the process of updating all parameters of a pretrained model on a specific task using labeled data, optimizing it for task-specific performance while retaining pretrained knowledge (Figure 1A). For encoder-based models, the original input is first processed by a tokenizer and then passed through the transformer embedding layer, producing initial token embeddings. These embeddings are static in the sense that they do not yet incorporate context. After passing through the transformer encoder layers, the model generates contextualized embeddings, where each token representation is influenced by its surrounding context. The hidden state of the [CLS] token is used as the sequence-level representation, and a classification layer is applied to this representation to predict the temporal relation class.

For decoder-based models, the input was formatted with a task-specific prompt to provide explicit instructions for temporal relation classification (Figure 3). The original input is structured as: “System Message + ‘Text: ’+ Original Input + ‘The temporal relationship between the subject and object is? <Answer>’.” The system message provides detailed instructions to guide the model’s response. For these models, the final hidden state of the last token was used as the sequence-level representation, and a classification layer was applied to this representation to predict the temporal relation class. Although the input was prompt-formatted, the decoder-based models were not trained as generative classifiers. Constrained decoding or free-text generation was not used to produce label tokens.

For both encoder-based and decoder-based models, the classification head produced logits over the three temporal relation classes, which were encoded as integer labels using the mapping BEFORE=0, AFTER=1, and OVERLAP=2. The models were trained using cross-entropy loss, and the predicted class was determined by the class with the highest softmax probability.

In standard fine-tuning, the model remains unfrozen, allowing all layers to be updated throughout the entire fine-tuning process. In this study, standard fine-tuning was applied exclusively to GatorTron-Base, as the other LLMs used—GatorTron-Large, LLaMA3.1-8B, and MeLLaMA-13B—were too large to fine-tune without freezing internal model parameters.

**Figure 3.**
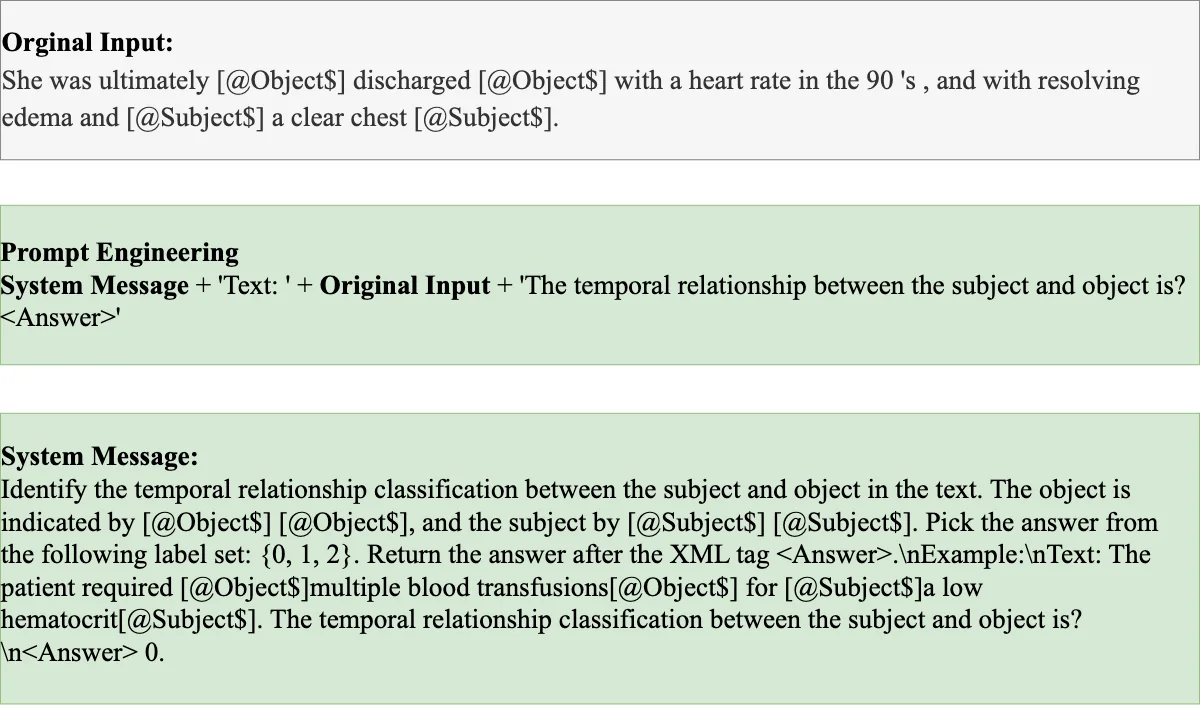
The prompt engineering for the transformer decoder-based models.

#### Hard-Prompting

Hard-prompting uses a structured prompt template, formatted as “Subject [MASK] [MASK] Object,” appended to the original input text (Figure 1B). By integrating this template, the CTRE task is reformulated into a MLM problem. The model is provided with predefined label words corresponding to each temporal relation class, such as “BEFORE” (labeled as “happened before”), “OVERLAP” (labeled as “happened overlap”), and “AFTER” (labeled as “happened after”). Rather than directly predicting the temporal relation, the model is used to predict the most probable label words to fill the [MASK] positions. The final predicted label words are then mapped to the corresponding temporal relation classification.

Specifically, the contextual vector is first derived by processing the original input text along with the appended hard-prompt template. This process yields the contextual representation of the [MASK] positions by selecting the corresponding slice from the overall contextual vector. Simultaneously, embedding vectors are derived for the predefined label words. The final classification is determined through a dot-product operation between the contextual vectors of the masked tokens and the embeddings of the predefined label words. This operation generates logits for each label word, allowing for the selection of the label word with the highest probability. The selected label word is then mapped to the corresponding temporal relation. Hard-prompting was applied exclusively to encoder-based models, as its underlying principle involves reformulating downstream tasks to align with the pretraining task format of encoder-based architectures, thereby enhancing the performance of pretrained language models (PLMs). Our previous study [51-53] demonstrated that reformatting downstream tasks to align with a model’s pretraining format significantly enhances performance. Additionally, for hard-prompting, we also kept the LLM weights unfrozen, allowing the model’s parameters to be adjusted during training to optimize task performance. Therefore, hard-prompting was implemented only for GatorTron-Base, as GatorTron-Large was too large to fine-tune without freezing internal model parameters.

#### Soft-Prompting

Soft-prompting is a technique that introduces learnable prompt embeddings, which are inserted into the input representation and processed alongside the original input by the frozen transformer encoder layers [54] (Figure 2A). This method facilitates task-specific adaptation without altering the pretrained model parameters, thereby substantially reducing computational overhead, as only the prompt embeddings are updated during fine-tuning.

#### LoRA

LoRA is a technique that freezes the pretrained model weights while introducing trainable rank decomposition matrices into each layer of the transformer architecture, significantly reducing the number of trainable parameters required for downstream tasks (Figure 2B). For instance, compared with fine-tuning GPT-3 175B using the Adam optimizer, LoRA can decrease the number of trainable parameters by 1000 times and reduce GPU memory requirements by threefold. Despite having fewer trainable parameters, LoRA achieves comparable or superior performance to standard fine-tuning [36]. In this study, we investigated the LoRA method to reduce trainable parameters, enabling the model to adapt to the CTRE task with minimal alteration to the PLMs.

### Experimental Settings

This study examines fine-tuning approaches under computational resource constraints. Given that GatorTron-Base has 345M parameters, full parameter fine-tuning was feasible and applied to this model. However, for GatorTron-Large (8.9B parameters), LLaMA3.1-8B (8B parameters), and MeLLaMA-13B (13B parameters), full parameter fine-tuning was impractical due to their large parameter sizes and the limitations of available computational resources. Consequently, only PEFT strategies were used for these models. As summarized in Table 3, GatorTron-Base was fine-tuned using the following 4 strategies: (1) standard fine-tuning, (2) hard-prompting, (3) soft-prompting, and (4) LoRA. In contrast, GatorTron-Large, LLaMA3.1-8B, and MeLLaMA-13B were fine-tuned using only soft-prompting and LoRA to ensure computational efficiency while adapting the models effectively.

**Table 3.** Fine-tuning strategies applied to different models in this study^a^.

Models	Standard fine-tuning	Hard-prompting	Soft-prompting	LoRA^b^
GatorTron-Base	✔	✔	✔	✔
GatorTron-Large			✔	✔
LLaMA3.1-8b			✔	✔
MeLLaMA-13b			✔	✔

a Quantization was used together with PEFT strategies, including soft-prompting and LoRA, whereas full-parameter fine-tuning strategies, including standard fine-tuning and hard-prompting, were conducted without quantization. QLoRA denotes LoRA applied to quantized models.

b LoRA: low-rank adaptation.

In this paper, we used 4-bit floating-point (NF4) quantization to load LLMs for reducing memory and computational demands by representing model weights and activations with lower-precision data types, enabling efficient deployment of larger models and faster inference [55]. In this study, quantization was used together with PEFT strategies, including soft-prompting and LoRA, whereas full-parameter fine-tuning strategies, including standard fine-tuning and hard-prompting, were conducted without quantization. The LoRA strategy with quantization is referred to as QLoRA. In QLoRA, LoRA weights are incorporated into specific layers of the quantized model [56].

For soft-prompting with LLMs, we built upon insights from previous studies that demonstrated optimal performance for soft-prompt mechanisms with token lengths of 32 and 64 [28]. Accordingly, we evaluated soft-prompts of these 2 lengths in our experiments. Additionally, we applied LoRA in 2 distinct configurations: one targeting all linear layers and another restricted specifically to the query and value layers within the attention mechanism. For transformer decoder-based models, the “all linear layers” configuration targets the *q_proj*, *k_proj*, *v_proj*, *o_proj*, *gate_proj*, *up_proj*, and *down_proj* modules. In the restricted configuration, LoRA is applied solely to the *q_proj* and *v_proj* modules. For transformer encoder-based models, the “all linear layers” configuration encompasses the *query*, *key*, *value*, and *dense* modules, whereas the restricted configuration targets only the *query* and *value* modules. For QLoRA, we used the same core LoRA hyperparameters for both the QLoRA (qv) and QLoRA (all linear layers) configurations to ensure a controlled comparison. Specifically, the LoRA rank was set to 16, the LoRA α was set to 32, and the LoRA dropout rate was set to 0.1.

To ensure the validity of model comparisons, identical hardware configurations were used across all experimental settings. Specifically, all experiments were conducted using H100 GPUs.

In this study, we examine the performance of models for 1-shot learning settings in addition to a data-rich setting. In the K-shot scenarios, “K” instances were selected from each relation type in the original complete training dataset to construct the corresponding few-shot training set. Specifically, in the 1-shot scenario, one instance was sampled from each relation type and used as the training data. For validation, we used the same validation dataset as that in the data-rich setting. This design provided a sufficiently large validation set for model selection. To ensure a consistent benchmark, the evaluation was conducted on the original, unmodified test set, identical to that used in the standard fully supervised scenario.

In the data-rich setting, models were trained for up to 10 epochs with an early stopping patience of 3 epochs; if convergence was not achieved, training was extended to 20 epochs with the same early stopping patience. In the few-shot setting, training was initially performed for 20 epochs with an early stopping patience of 3 epochs, and if the model did not converge, training continued for up to 30 epochs. The learning rate was set according to model architecture. Encoder-based models were trained with a learning rate of 1e-4, whereas decoder-based models were trained with a learning rate of 2e-4. These architecture-specific learning rates were selected to support stable optimization across different backbone architectures.

For the data-rich regime, batch size and gradient accumulation steps were adjusted according to model architecture, adaptation strategy, and GPU memory requirements. Decoder-based QLoRA, decoder-based soft-prompting, and encoder-based standard fine-tuning used a training batch size of 4 with gradient accumulation steps of 4. Encoder-based QLoRA used a training batch size of 16 with gradient accumulation steps of 2. Encoder-based hard-prompting and encoder-based soft-prompting used a training batch size of 16 with gradient accumulation steps of 4. The settings were selected based on GPU memory requirements and training feasibility. Decoder-based models and full standard fine-tuning required substantially more GPU memory, so smaller per-device batch sizes were necessary. In contrast, encoder-based PEFT configurations were less memory intensive and allowed larger batch sizes. These settings were chosen to ensure feasible and stable training under GPU memory constraints. Because memory consumption differed substantially across architectures and adaptation strategies, identical batch settings were not always feasible. Therefore, we transparently report these settings and interpret cross-model comparisons cautiously, focusing on matched adaptation strategies, comparable trainable-parameter groups, and repeated-run mean (SD) performance. In addition, the approximate training time for each experiment has been provided in Multimedia Appendix 1.

To improve the robustness of the experimental results, each experimental setting was repeated using 3 random seeds: 1234, 42, and 47. For each setting, we reported the mean and SD of micro-*F*_1_, macro-*F*_1_, and weighted-*F*_1_ across the three runs. Micro-*F*_1_ was used as the primary evaluation metric, as it reflects the overall classification performance across all instances and provides an aggregate measure of model accuracy across relation types. Given that the dataset was imbalanced across relation classes, macro-*F*_1_ and weighted-*F*_1_ were also reported as supplementary metrics. Macro-*F*_1_ assigns equal importance to each class and is therefore useful for assessing model performance on minority classes, whereas weighted-*F*_1_ accounts for class support and reflects performance adjusted by the distribution of relation types. In addition, class-wise precision, recall, and *F*_1_-scores were calculated, and error analysis was conducted to further examine model behavior across relation types.

### External Validation

This study was designed as a comparative analysis of multiple language models; we saved the best-performing fine-tuned checkpoint for each model under the data-rich regime on the 2012 i2b2 CTRE dataset and applied these checkpoints to the MACCROBAT [57,58] subset for external validation. This setting allowed us to examine whether the relative performance patterns observed on the i2b2 benchmark were maintained on an external dataset with a different source and annotation context.

MACCROBAT consists of 200 clinical case reports from PubMed Central and contains 19,089 annotated clinical events. Although the dataset was originally developed for broader clinical information extraction tasks, it also includes temporal relations annotated with three relation categories: BEFORE, AFTER, and OVERLAP. For external validation, we extracted a balanced subset of 1000 temporal relation instances from MACCROBAT, including 333 BEFORE instances, 334 AFTER instances, and 333 OVERLAP instances.

### Ethical Considerations

This study used 2 previously released, deidentified benchmark datasets—the 2012 i2b2 temporal relations challenge corpus and MACCROBAT. The i2b2 dataset was accessed and used in accordance with its Data Use Agreement, and MACCROBAT is based on publicly available clinical case reports from PubMed Central. No new human participant data were collected, and no direct patient identifiers were accessed. Therefore, additional institutional review board approval and informed consent were not required.

## Results

### Data-Rich Setting

QLoRA (qv) refers to QLoRA adaptation applied only to the query and value projection layers, whereas QLoRA (all) refers to QLoRA adaptation applied to all linear layers. Table 4 compares 2 QLoRA fine-tuning strategies, QLoRA (qv) and QLoRA (all), using micro-*F*_1_-scores reported as mean (SD) percentages across 3 runs. Overall, both strategies achieved competitive performance, and the relative advantage varied across models. For LLaMA3.1-8B, QLoRA (qv) achieved a higher micro-*F*_1_-score than QLoRA (all), with 89.15% (0.30) compared with 88.30% (1.18). In contrast, MeLLaMA-13B showed a slightly higher score when all linear layers were fine-tuned, with 88.94% (0.28) compared with 88.04% (2.15) for QLoRA (qv). For GatorTron-Large, the 2 strategies produced nearly identical results, with 87.33% (0.19) for QLoRA (qv) and 87.31% (0.27) for QLoRA (all). Similarly, for GatorTron-Base, QLoRA (qv) achieved 87.22% (0.65), while QLoRA (all) achieved 87.62% (0.24).

Across the 4 models, QLoRA (qv) achieved the highest micro-*F*_1_-score for LLaMA3.1-8B, whereas QLoRA (all) achieved slightly higher scores for MeLLaMA-13B and GatorTron-Base. The difference between the 2 strategies was minimal for GatorTron-Large. These results indicate that fine-tuning all linear layers did not consistently improve micro-*F*_1_ performance over fine-tuning only the query and value projection layers in the data-rich setting.

**Table 4.** Comparison between two QLoRA^a^ fine-tuning strategies: QLoRA (qv)^b^ and QLoRA (all)^c^.

Models	QLoRA (qv)^d^	QLoRA (all)^d^
LLaMA3.1-8B	89.15 (0.30)^e^	88.30 (1.18)
MeLLaMA-13B	88.04 (2.15)	88.94 (0.28)^e^
GatorTron-Large	87.33 (0.19)^e^	87.31 (0.27)
GatorTron-Base	87.22 (0.65)	87.62 (0.24)^e^

a QLoRA: quantized low-rank adaptation.

b QLoRA (qv) refers to QLoRA adaptation applied only to the query and value projection layers.

c QLoRA (all) refers to QLoRA adaptation applied to all linear layers.

d Micro-*F*_1_-scores are reported as mean (SD) percentages across 3 runs.

e Better-performing result between the two QLoRA fine-tuning strategies for each model.

Table 5 compares decoder-based and encoder-based LLMs under settings with broadly comparable numbers of trainable parameters. Specifically, LLaMA3.1-8B (QLoRA qv: 7M), MeLLaMA-13B (QLoRA qv: 13M), GatorTron-Large (QLoRA qv: 13M), and GatorTron-Base (QLoRA all: 7M) have similar trainable parameter sizes. Likewise, LLaMA3.1-8B (QLoRA all: 42M), MeLLaMA-13B (QLoRA all: 62M), and GatorTron-Large (QLoRA all: 58M) form another comparable group. The results are reported as mean (SD) percentages across 3 runs, including micro-*F*_1_, macro-*F*_1_, and weighted-*F*_1_.

In group A, the trainable parameter sizes ranged from 7M to 13M. LLaMA3.1-8B with QLoRA (qv) achieved the highest micro-*F*_1_-score in this group, with 89.15% (0.30), followed by MeLLaMA-13B with QLoRA (qv), which achieved 88.04% (2.15). The encoder-based GatorTron models achieved slightly lower micro-*F*_1_-scores, with 87.33% (0.19) for GatorTron-Large with QLoRA (qv) and 87.62% (0.24) for GatorTron-Base with QLoRA (all). A similar pattern was observed for weighted-*F*_1_, where LLaMA3.1-8B achieved 89.08% (0.30), followed by MeLLaMA-13B at 87.97% (2.21), GatorTron-Base at 87.50% (0.21), and GatorTron-Large at 87.11% (0.32).

In group B, which included models with larger numbers of trainable parameters, MeLLaMA-13B with QLoRA (all) achieved the highest micro-*F*_1_-score, with 88.94% (0.28), followed by LLaMA3.1-8B with QLoRA (all), which achieved 88.30% (1.18). GatorTron-Large with QLoRA (all) achieved a micro-*F*_1_-score of 87.31% (0.27). The same general trend was observed for macro-*F*_1_ and weighted-*F*_1_. MeLLaMA-13B achieved the highest macro-*F*_1_ and weighted-*F*_1_-scores in group B, with 84.1% (0.55) and 88.87% (0.32), respectively, while GatorTron-Large achieved 81.15% (0.22) for macro-*F*_1_ and 87.17% (0.25) for weighted-*F*_1_.

Overall, within these comparable trainable-parameter groups, the decoder-based models generally achieved higher performance than the encoder-based GatorTron models.

**Table 5.** Comparison of decoder-based and encoder-based LLMs^a^ with comparable trainable parameters.

Models	Trainable parameters	Micro-*F*_1_, mean (SD)	Macro-*F*_1_, mean (SD)	Weighted-*F*_1_, mean (SD)
Group A				
LLaMA3.1-8b_QLoRA^b^ (qv)^c^	7M	89.15 (0.30)	84.44 (0.33)	89.08 (0.30)
MeLLaMA-13b_QLoRA (qv)	13M	88.04 (2.15)	82.69 (3.42)	87.97 (2.21)
GatorTron-Large_QLoRA (qv)	13M	87.33 (0.19)	81.00 (0.60)	87.11 (0.32)
GatorTron-Base_QLoRA (all)^d^	7M	87.62 (0.24)	81.76 (0.30)	87.50 (0.21)
Group B				
LLaMA3.1-8b_QLoRA (all)	42M	88.30 (1.18)	83.39 (1.30)	88.33 (1.03)
MeLLaMA-13b_QLoRA (all)	62M	88.94 (0.28)	84.10 (0.55)	88.87 (0.32)
GatorTron-Large_QLoRA (all)	58M	87.31 (0.27)	81.15 (0.22)	87.17 (0.25)

a LLM: large language model.

b QLoRA: quantized low-rank adaptation.

c QLoRA (qv) refers to QLoRA adaptation applied only to the query and value projection layers.

d QLoRA (all) refers to QLoRA adaptation applied to all linear layers.

Table 6 compares standard fine-tuning and hard-prompting on GatorTron-Base using micro-*F*_1_, macro-*F*_1_, and weighted-*F*_1_-scores reported as mean (SD) percentages across 3 runs. Both strategies used the same number of trainable parameters, 345M. Standard fine-tuning and hard-prompting achieved comparable micro-*F*_1_-scores, with 86.86% (0.44) and 86.1% (0.61), respectively. For macro-*F*_1_, the 2 strategies obtained 80.49% (0.65) and 80.17% (1.04), respectively. A consistent pattern was observed for weighted-*F*_1_, with standard fine-tuning achieving 86.68% (0.44) and hard-prompting achieving 86% (0.63). Overall, the 2 strategies produced broadly comparable results across all evaluation metrics.

**Table 6.** Comparison between hard-prompting and standard fine-tuning in GatorTron-Base models.

Fine-tuning strategies	Trainable parameters	Micro-*F*_1_, mean (SD)	Macro-*F*_1_, mean (SD)	Weighted-*F*_1_, mean (SD)
Standard fine-tuning	345M	86.86 (0.44)	80.49 (0.65)	86.68 (0.44)
Hard-prompting	345M	86.10 (0.61)	80.17 (1.04)	86.00 (0.63)

Table 7 presents the performance of the soft-prompting strategy across different LLMs with prompt lengths of 32 and 64. Micro-*F*_1_, macro-*F*_1_, and weighted-*F*_1_-scores are reported as mean (SD) percentages across 3 runs. Under the soft-prompting setting, the encoder-based GatorTron models achieved higher performance than the decoder-based LLaMA3.1-8B and MeLLaMA-13B models. Specifically, GatorTron-Base achieved micro-*F*_1_-scores of 71.07% (0.15) and 72.88% (0.60) with prompt lengths of 32 and 64, respectively, while GatorTron-Large achieved 71.65% (0.46) and 70.63% (1.06). In contrast, the decoder-based models showed substantially lower micro-*F*_1_-scores. LLaMA3.1-8B achieved 49.32% (0.30) with a prompt length of 32 and 49.31% (0.18) with a prompt length of 64, whereas MeLLaMA-13B achieved 49.47% (0.42) and 50.01% (0.71), respectively. A similar pattern was observed for macro-*F*_1_ and weighted-*F*_1_, with the encoder-based models consistently outperforming the decoder-based models across both prompt lengths.

The effect of prompt length was not consistent across models. For GatorTron-Base, increasing the prompt length from 32 to 64 improved micro-*F*_1_ from 71.07% (0.15) to 72.88% (0.60). However, for GatorTron-Large, the prompt length of 32 produced a higher micro-*F*_1_-score than the prompt length of 64. For LLaMA3.1-8B, the 2 prompt lengths resulted in nearly identical micro-*F*_1_-scores, with 49.32% (0.30) for prompt length 32 and 49.31% (0.18) for prompt length 64. For MeLLaMA-13B, the 2 prompt lengths also resulted in similar micro-*F*_1_-scores. These results indicate that neither prompt length showed a consistent advantage across all models.

Overall, soft-prompting achieved lower performance than the other fine-tuning strategies evaluated in this study, although it required substantially fewer trainable parameters. These results suggest that soft-prompting was less effective for CTRE in the current experimental setting, particularly for decoder-based models.

**Table 7.** Performance of soft-prompting across different LLMs^a^ with prompt lengths of 32 and 64.

Models (quantized) and soft-prompt length	Trainable parameters	Micro-*F*_1_, mean (SD)	Macro-*F*_1_, mean (SD)	Weighted-*F*_1_, mean (SD)
GatorTron-Base				
32	36K	71.07 (0.15)	49.24 (0.13)	67.62 (0.15)
64	69K	72.88 (0.60)	50.60 (0.43)	69.40 (0.57)
GatorTron-Large				
32	125K	71.65 (0.46)	49.63 (0.38)	68.17 (0.48)
64	240K	70.63 (1.06)	48.67 (0.93)	67.00 (1.16)
LLaMA3.1-8B				
32	143K	49.32 (0.30)	35.35 (1.49)	44.43 (0.40)
64	274K	49.31 (0.18)	35.86 (1.91)	44.39 (0.69)
MeLLaMA-13B				
32	179K	49.47 (0.42)	35.67 (2.10)	44.23 (1.21)
64	343K	50.01 (0.71)	36.15 (0.85)	45.19 (1.15)

a LLM: large language model.

To further contextualize the results in the data-rich regime, we compared our findings with previously reported performance on the 2012 i2b2 CTRE benchmark. Knez and Žitnik [23] evaluated their model using the predefined test portion of the benchmark dataset, which is consistent with the evaluation protocol adopted in our study. Under this comparable setting, their highest reported micro-*F*_1_-score was 82.04%. In comparison, several configurations in our study achieved higher micro-*F*_1_-scores. The best-performing configuration was LLaMA3.1-8B with QLoRA (qv), which achieved 89.15% (0.30), followed by MeLLaMA-13B with QLoRA (all) at 88.94% (0.28). LLaMA3.1-8B with QLoRA (all), MeLLaMA-13B with QLoRA (qv), and GatorTron-Large with QLoRA (qv) also achieved strong results, with micro-*F*_1_-scores of 88.3% (1.18), 88.04% (2.15), and 87.33% (0.19), respectively. GatorTron-Base with standard fine-tuning and hard-prompting also performed strongly, achieving micro-*F*_1_-scores of 86.72% (0.19) and 86.1% (0.61), respectively.

Nevertheless, comparisons with previous studies should still be interpreted cautiously because evaluation settings are not always fully aligned across studies. For example, Uma et al [24] reported a micro-*F*_1_-score of 85.7%, but their study used a custom 80/10/10 data split and explicitly noted that their results were not directly comparable with earlier i2b2 studies because of differences in relation scope, data splitting, and evaluation settings. Although several configurations in our study achieved numerically higher micro-*F*_1_-scores than this previously reported result, these differences should be interpreted in light of the distinct experimental settings. Therefore, rather than claiming a definitive new SOTA result, we interpret our findings as providing strong contextualized evidence that recent LLMs combined with appropriate adaptation strategies can achieve competitive performance on the 2012 i2b2 CTRE benchmark.

### Error Analysis

To further examine model behavior beyond aggregate micro-*F*_1_, we conducted an error analysis using the best-performing configuration in the data-rich regime. Specifically, LLaMA3.1-8B with QLoRA (qv) achieved the highest overall performance among the evaluated configurations, with a mean micro-*F*_1_-score of 89.15% (SD 0.30) across 3 runs. Among the 3 random seeds, seed 47 achieved the highest single-run result; therefore, this run was used for detailed error analysis. On the predefined test set, this model achieved a micro-*F*_1_-score of 89.4%, a macro-*F*_1_-score of 84.69%, and a weighted-*F*_1_-score of 89.34%. The confusion matrix was illustrated in Table 8, where the rows represent gold labels and the columns represent predicted labels in the order of BEFORE, AFTER, and OVERLAP. Overall, 2940 of 27,736 test instances were misclassified.

**Table 8.** Confusion matrix of the best-performing configuration in the data-rich regime.

Gold label \ Predicted label^a^	BEFORE	AFTER	OVERLAP	Total
BEFORE	13,962	247	904	15,113
AFTER	312	1889	528	2729
OVERLAP	623	326	8945	9894
Total	14,897	2462	10,377	27,736

a Rows represent gold labels, and columns represent predicted labels. The diagonal values indicate correctly classified instances.

Table 9 presents the per-class precision, recall, and *F*_1_-score for this configuration. BEFORE achieved the highest class-level performance, with a precision of 93.72%, recall of 92.38%, and *F*_1_-score of 93.05%. OVERLAP also showed strong performance, with a precision of 86.20%, recall of 90.41%, and *F*_1_-score of 88.25%. In contrast, AFTER was the most challenging class, with a precision of 76.73%, recall of 69.22%, and *F*_1_-score of 72.78%. This difference explains why macro-*F*_1_ was lower than micro-*F*_1_ and weighted-*F*_1_. Because the dataset is imbalanced, micro-*F*_1_ and weighted-*F*_1_ mainly reflect overall performance across all instances and are more influenced by the majority classes, whereas macro-*F*_1_ gives equal weight to each class and is more sensitive to the lower performance on the minority AFTER class.

Qualitative inspection of the misclassified cases suggested 2 major error characteristics. The dominant error pattern was the confusion between BEFORE and OVERLAP. Specifically, 904 BEFORE instances were incorrectly classified as OVERLAP, accounting for 30.75% (904/2940) of all misclassified cases, while 623 OVERLAP instances were incorrectly classified as BEFORE, accounting for 21.19% (623/2940) of all errors. These 2 directions together represented more than half (1527/2940, 51.94%) of all misclassifications (1527 of 2940 misclassified cases).

A representative example is “Workup on admission included [@Object$]a chest x-ray[@Object$] negative for [@Subject$]pneumonia[@Subject$], an MRI of the liver to investigate for focal lesion, with no evidence for abscess or focal lesion on report.” In this case, the gold label was BEFORE, but the model predicted OVERLAP. The diagnostic test and the clinical condition were semantically related and appeared in close textual proximity. The model may therefore have interpreted their clinical association as temporal overlap, even though the gold annotation indicated a sequential relation.

Additionally, the model showed greater difficulty in identifying AFTER relations. This is consistent with the per-class results, where AFTER had the lowest precision, recall, and *F*_1_-score among the 3 relation types. Among the true AFTER instances, 312 were predicted as BEFORE, and 528 were predicted as OVERLAP. A representative example is “She was [@Object$]discharged[@Object$] on [@Subject$]Coumadin[@Subject$] for a goal INR of 2.5 to 3,” where the gold label was AFTER, but the model predicted BEFORE. This example illustrates the difficulty of resolving temporal relations in compressed discharge statements. In clinical documentation, expressions such as “discharged on” may encode not only the discharge event itself but also an ongoing or postdischarge treatment plan. Therefore, correctly identifying the relation requires interpreting the implicit temporal scope of the clinical event, rather than relying only on the local word order of the sentence.

Overall, these findings suggest that the remaining errors were mainly concentrated in context-dependent temporal reasoning cases. In clinical narratives, annotated events may be semantically related and textually close, but their temporal relation may still depend on broader information from surrounding sentences, the hospital course, or the full discharge summary. Thus, although the best-performing QLoRA-based LLaMA3.1-8B model captured explicit temporal relations effectively, it was less robust when local textual evidence was insufficient. The main residual challenges involved identifying AFTER relations and distinguishing sequential relations from overlapping relations under limited local temporal cues.

**Table 9.** Per-class performance of the best-performing configuration in the data-rich regime.

Class	Precision	Recall	*F*_1_-score
BEFORE	93.72	92.38	93.05
AFTER	76.73	69.22	72.78
OVERLAP	86.20	90.41	88.25
Macro average	85.88	84.00	84.69
Weighted average	89.31	89.40	89.34

### Few-Shot Scenarios

Tables 10–13 present the results in the 1-shot scenario. In the comparison between QLoRA (qv) and QLoRA (all), fine-tuning all linear layers did not consistently improve performance across models. Although QLoRA (all) achieved higher micro-*F*_1_-scores for LLaMA3.1-8B and MeLLaMA-13B, QLoRA (qv) remained competitive for GatorTron-Large and produced nearly identical performance for GatorTron-Base. Considering that QLoRA (qv) used fewer trainable parameters, these results suggest that it remained a computationally efficient and competitive adaptation strategy under limited supervision. Similarly, when models with broadly comparable numbers of trainable parameters were compared, decoder-based models generally achieved stronger performance than encoder-based models under QLoRA-based adaptation, consistent with the trend observed in the data-rich setting.

Hard-prompting achieved better performance than standard fine-tuning in the 1-shot scenario, with higher micro-*F*_1_, macro-*F*_1_, and weighted-*F*_1_-scores. Under the more parameter-efficient soft-prompting setting, encoder-based GatorTron models outperformed decoder-based LLaMA3.1-8B and MeLLaMA-13B models, with GatorTron-Large using a prompt length of 64 achieving the strongest soft-prompting performance.

**Table 10.** Comparison between 2 QLoRA^a^ fine-tuning strategies: QLoRA (qv)^b^ and QLoRA (all)^c^ for 1-shot scenario.

Models	QLoRA (qv)^d^	QLoRA (all)^d^
LLaMA3.1-8B	51.96 (2.01)	55.99 (2.56)
MeLLaMA-13B	44.81 (7.85)	48.18 (10.04)
GatorTron-Large	41.42 (9.94)	39.42 (11.58)
GatorTron-Base	45.70 (11.69)	45.75 (11.80)

a QLoRA: quantized low-rank adaptation.

b QLoRA (qv) refers to QLoRA adaptation applied only to the query and value projection layers.

c QLoRA (all) refers to QLoRA adaptation applied to all linear layers.

d Micro-*F*_1_-scores are reported as mean (SD) percentages across 3 runs.

**Table 11.** Comparison of decoder-based and encoder-based LLMs^a^ with comparable trainable parameters for 1-shot scenario.

Models	Trainable parameters	Micro-*F*_1_, mean (SD)	Macro-*F*_1_, mean (SD)	Weighted-*F*_1_, mean (SD)
Group A				
LLaMA3.1-8b_QLoRA^b^ (qv)^c^	7M	51.96 (2.01)	28.94 (5.77)	41.49 (4.25)
MeLLaMA-13b_QLoRA (qv)	13M	44.81 (7.85)	23.94 (4.19)	33.95 (9.83)
GatorTron-Large_QLoRA (qv)	13M	41.42 (9.94)	23.15 (4.15)	31.79 (8.60)
GatorTron-Base_QLoRA (all)^d^	7M	45.75 (11.80)	23.62 (5.30)	33.30 (10.58)
Group B				
LLaMA3.1-8b_QLoRA (all)	42M	55.99 (2.56)	28.42 (8.36)	43.69 (8.95)
MeLLaMA-13b_QLoRA (all)	62M	48.18 (10.04)	21.92 (2.66)	32.48 (10.06)
GatorTron-Large_QLoRA (all)	58M	39.42 (11.58)	23.94 (5.36)	31.24 (8.31)

a LLM: large language model.

b QLoRA: quantized low-rank adaptation.

c QLoRA (qv) refers to QLoRA adaptation applied only to the query and value projection layers.

d QLoRA (all) refers to QLoRA adaptation applied to all linear layers.

**Table 12.** Comparison between hard-prompting and standard fine-tuning for 1-shot scenario for GatorTron-Base model.

Fine-tuning strategies	Trainable parameters	Micro-*F*_1_, mean (SD)	Macro-*F*_1_, mean (SD)	Weighted-*F*_1_, mean (SD)
Standard fine-tuning	345M	54.08 (0.71)	23.84 (0.58)	38.40 (0.08)
Hard-prompting	345M	58.17 (4.22)	34.29 (8.01)	49.46 (9.38)

**Table 13.** Performance of soft-prompting across different LLMs^a^ with prompt lengths of 32 and 64 for 1-shot scenario.

Models (quantized) and soft-prompt length	Trainable parameters	Micro-*F*_1_, mean (SD)	Macro-*F*_1_, mean (SD)	Weighted-*F*_1_, mean (SD)
GatorTron-Base				
32	36K	43.67 (9.57)	24.43 (3.94)	36.57 (3.98)
64	69K	47.81 (8.14)	24.75 (3.93)	37.71 (4.03)
GatorTron-Large				
32	125K	44.81 (8.59)	25.38 (4.92)	37.34 (3.67)
64	240K	56.25 (3.54)	32.43 (7.01)	46.89 (7.71)
LLaMA3.1-8B				
32	143K	34.86 (5.40)	25.18 (2.93)	27.11 (7.95)
64	274K	32.6 (3.00)	21.19 (0.98)	21.04 (2.68)
MeLLaMA-13B				
32	179K	33.29 (4.79)	24.03 (1.74)	25.8 (6.82)
64	343K	33.9 (4.65)	24.75 (2.70)	26.9 (6.86)

a LLM: large language model.

### External Validation Results

Table 14 presents the external validation results using checkpoints trained in the data-rich regime with seed 42 and evaluated directly on an independent CTRE dataset, MACCROBAT. As expected in a cross-dataset setting, the absolute performance was lower than that observed on the main 2012 i2b2 benchmark, which may reflect differences in document source, annotation practice, relation distribution, and linguistic characteristics. Nevertheless, the external validation results partially supported the main findings regarding QLoRA-based adaptation. QLoRA (qv) remained competitive with QLoRA applied to all linear layers, suggesting that adapting only the query and value projection layers can provide a computationally efficient strategy without substantially compromising external validation performance. In addition, decoder-based models, particularly LLaMA3.1-8B, achieved strong transfer performance under QLoRA-based adaptation. Taken together, these findings suggest that the main conclusions from the data-rich experiments were partially preserved under external validation: QLoRA (qv) remained an efficient and competitive adaptation strategy, and decoder-based models, particularly LLaMA3.1-8B, showed strong cross-dataset transferability.

The detailed results, including the seed-specific micro-*F*_1_, macro-*F*_1_, and weighted-*F*_1_-scores for each configuration in both the data-rich and 1-shot regimes, as well as the per-class performance metrics, are provided in Multimedia Appendix 1.

**Table 14.** External validation results on the MACCROBAT dataset.

Model	Adaptation strategy	Micro-*F*_1_^a^	Macro-*F*_1_^a^	Weighted-*F*_1_^a^
LLaMA3.1-8B	QLoRA^b^ (all)^c^	62.40	61.59	61.58
LLaMA3.1-8B	QLoRA (qv)^d^	61.60	60.43	60.42
MeLLaMA-13B	QLoRA (all)	56.80	54.37	54.35
MeLLaMA-13B	QLoRA (qv)	49.80	46.56	46.55
GatorTron-Base	QLoRA (all)	59.80	57.76	57.74
GatorTron-Base	QLoRA (qv)	61.00	59.58	59.57
GatorTron-Large	QLoRA (all)	56.90	54.24	54.22
GatorTron-Large	QLoRA (qv)	57.30	53.90	53.88
GatorTron-Base	Standard_Fine-Tuning	48.20	44.31	44.30
GatorTron-Base	Hard-Prompting	51.40	48.05	48.03

a Micro-*F*_1_, macro-*F*_1_, and weighted-*F*_1_-scores are reported as mean percentages.

b QLoRA: quantized low-rank adaptation.

c QLoRA (all) refers to QLoRA adaptation applied to all linear layers.

d QLoRA (qv) refers to QLoRA adaptation applied only to the query and value projection layers.

## Discussion

This study systematically evaluated the performance of different language models and adaptation strategies for CTRE. In the data-rich setting, multiple adaptation strategies achieved strong performance on the 2012 i2b2 CTRE benchmark. The best-performing configuration was LLaMA3.1-8B with QLoRA applied to the query and value projection layers, which achieved a micro-*F*_1_-score of 89.15% (0.30). In addition, other QLoRA-based configurations, standard fine-tuning, and hard-prompting also produced strong results. For example, MeLLaMA-13B with QLoRA (all) achieved 88.94% (0.28), while LLaMA3.1-8B with QLoRA (all) achieved 88.3% (1.18). Standard fine-tuning and hard-prompting on GatorTron-Base also achieved competitive micro-*F*_1_-scores of 86.86% (0.44) and 86.1% (0.61), respectively. Compared with previously reported results on the 2012 i2b2 CTRE dataset, these findings provide contextualized evidence that recent LLMs combined with appropriate adaptation strategies can achieve strong performance for CTRE. However, because previous studies differ in data splits, relation scope, and evaluation protocols, these results should not be interpreted as a definitive new SOTA claim. Instead, this study provides a controlled benchmark that demonstrates how different adaptation strategies and model architectures influence CTRE performance under varying data and parameter constraints.

The comparison between QLoRA (qv) and QLoRA (all) showed that increasing the number of trainable LoRA modules did not consistently improve performance. In the data-rich setting, QLoRA (qv) achieved the best overall result for LLaMA3.1-8B, whereas QLoRA (all) produced slightly higher micro-*F*_1_-scores for MeLLaMA-13B and GatorTron-Base. A similar pattern was observed in the 1-shot scenario and external validation, where QLoRA (qv) remained competitive with QLoRA (all). These results suggest that adapting only the query and value projection layers can provide a computationally efficient strategy without substantially compromising performance. This finding is practically important because QLoRA (qv) updates fewer trainable parameters and therefore reduces computational requirements compared with adapting all linear layers. Nevertheless, the relative advantage varied across models, indicating that the optimal QLoRA configuration is model-dependent rather than universal.

The comparison between decoder-based and encoder-based models further showed that architectural advantages depended on both the adaptation strategy and the resource setting. Under QLoRA-based adaptation, decoder-based models generally achieved stronger performance than encoder-based GatorTron models when the number of trainable parameters was broadly comparable. This trend was observed in both the data-rich and 1-shot settings, suggesting that recent decoder-based LLMs can be highly effective for CTRE when sufficient parameter-efficient adaptation is allowed. This finding contrasts with earlier assumptions that encoder-based models are generally preferable for relation extraction and suggests that the strong language modeling capacity of modern decoder-based LLMs can transfer effectively to clinical relation classification when adapted appropriately.

However, this advantage did not hold under the most parameter-efficient soft-prompting setting. When both the amount of training data and the number of trainable parameters were highly limited, encoder-based GatorTron models outperformed decoder-based LLaMA3.1-8B and MeLLaMA-13B. This pattern was especially evident in the 1-shot soft-prompting results, where GatorTron-Large achieved the strongest performance among the evaluated soft-prompting configurations. One possible explanation is that soft-prompting updates only a small number of continuous prompt parameters, making performance more dependent on the intrinsic representations learned during pretraining. Because GatorTron was pretrained on large-scale clinical corpora, its representations may be better aligned with the clinical language and temporal reasoning patterns in i2b2. In contrast, decoder-based models may require more extensive parameter adaptation to fully exploit their capacity for CTRE.

The comparison between standard fine-tuning and hard-prompting on GatorTron-Base also showed that the effectiveness of hard-prompting depends on the data regime. In the data-rich setting, standard fine-tuning and hard-prompting achieved broadly comparable performance on GatorTron-Base. In contrast, in the 1-shot scenario, hard-prompting achieved better performance than standard fine-tuning across micro-*F*_1_, macro-*F*_1_, and weighted-*F*_1_ on GatorTron-Base. This suggests that explicitly reformulating the task through a prompt template may be beneficial when very limited supervision is available. The advantage of hard-prompting in the 1-shot setting may be related to its closer alignment with the masked language modeling objective of encoder-based models. However, hard-prompting also has practical limitations, as its performance can be sensitive to prompt design and may require domain expertise to construct effective templates. Therefore, while hard-prompting may be useful in extremely low-resource scenarios, its generalizability and ease of implementation should be considered carefully.

Soft-prompting showed limited effectiveness for CTRE compared with other fine-tuning strategies evaluated in this study. Although soft-prompting required substantially fewer trainable parameters, its performance was considerably lower in the data-rich setting across the evaluated models. One possible explanation is that the limited representational capacity of short continuous prompts may create an information bottleneck for CTRE, which requires modeling complex temporal dependencies between clinical events. In the 1-shot setting, soft-prompting was more informative for comparing model architectures under strict parameter constraints, but its overall performance remained lower than strategies involving more extensive parameter updates. However, this interpretation should be considered in light of the specific soft-prompting configurations tested in this study. We evaluated prompt lengths of 32 and 64, and it is possible that longer prompt lengths, alternative prompt initialization strategies, or other prompt-based PEFT methods such as prefix tuning may yield different results. Therefore, our findings should not be interpreted as a general conclusion about all prompt-based PEFT approaches, but rather as evidence that the soft-prompting configurations evaluated here were less effective for high-precision CTRE than QLoRA-based adaptation or standard fine-tuning.

The external validation on MACCROBAT provided a preliminary assessment of cross-dataset transferability. As expected, the absolute performance was lower than that observed on the main 2012 i2b2 benchmark, likely reflecting differences in document source, annotation practice, relation distribution, and linguistic characteristics. Nevertheless, the external validation results partially preserved the main findings from the data-rich experiments. QLoRA (qv) remained competitive with QLoRA (all), supporting its role as an efficient and competitive adaptation strategy. In addition, LLaMA3.1-8B showed strong transfer performance under QLoRA-based adaptation, suggesting that decoder-based models can retain good cross-dataset generalizability when appropriately fine-tuned. At the same time, the performance drop highlights the difficulty of transferring CTRE models across datasets with different document types and annotation characteristics.

This study has several limitations. First, although the main experiments were conducted on the widely used 2012 i2b2 CTRE benchmark and supplemented with external validation on MACCROBAT, the evaluation still covered a limited range of CTRE datasets. Different datasets may vary in document type, event density, temporal expression patterns, and annotation guidelines, which may affect both absolute performance and model ranking. Second, this study used a 3-class relation setting consisting of BEFORE, AFTER, and OVERLAP to support controlled comparison and external validation after label harmonization. Other CTRE datasets may use more fine-grained temporal relation schemas, such as temporal containment, simultaneity, or boundary-specific relations. These finer distinctions may introduce additional ambiguity and may especially affect macro-*F*_1_ and minority-class performance. Third, the evaluated decoder-based models were limited to parameter-constrained variants. Larger models or more advanced instruction-tuned clinical LLMs may yield different performance patterns, particularly in low-resource settings. Future work should evaluate a broader range of CTRE datasets, relation schemas, and model scales to further assess the robustness and generalizability of these findings.

In summary, this study provides a controlled comparative benchmark of 4 LLMs and multiple adaptation strategies for CTRE. The results show that recent LLMs combined with appropriate adaptation strategies can achieve strong performance in the data-rich setting, with QLoRA (qv) offering a competitive and computationally efficient alternative to QLoRA (all). Decoder-based models generally performed better under QLoRA-based adaptation when sufficient trainable parameters were available, whereas encoder-based models showed advantages under the strictest soft-prompting constraints. Hard-prompting was particularly effective in the 1-shot setting compared with standard fine-tuning, but its advantage was not universal across settings. Overall, these findings highlight the importance of jointly considering model architecture, adaptation strategy, data availability, and computational constraints when designing CTRE systems for clinical narratives.

## Supplementary material

10.2196/89957 Multimedia Appendix 1 Detailed results.

## Abbreviations

CNN: convolutional neural networks
CRF: conditional random fields
CTRE: clinical temporal relation extraction
DCT: document creation time
EHR: electronic health record
FPFT: full-parameter-fine-tuning
GQA: grouped query attention
IAA: interannotator agreement
LLM: large language model
LoRA: low-rank adaptation
LR: logistic regression
LSTM: long short-term memory
MIBE: medical evaluation benchmark
MLM: masked language modeling
NLP: natural language processing
PEFT: parameter efficient fine-tuning
PLM: pretrained language model
QLoRA: quantized low-rank adaptation
RF: random forest
RNN: recurrent neural networks
SOP: sentence-order prediction
SOTA: state of the art
SVM: support vector machine
TLINK: temporal link
TRE: temporal relation extraction
